# Pulmonary Actinomycosis Revealed by a Solitary Pulmonary Nodule

**DOI:** 10.1155/2022/7877729

**Published:** 2022-02-16

**Authors:** Deghdegh Khaled, Terra Besma, Amoura Kamel, Benali Rachid

**Affiliations:** ^1^Pulmonary Department Annaba University Hospital Center, Annaba, Algeria; ^2^Central Laboratory of Biology CAC University Hospital Center, Annaba, Algeria

## Abstract

**Background:**

Pulmonary actinomycosis (PA) is a rare and ubiquitous bacterial disorder, combining subacute or chronic focal suppuration and an expansive fibrogranulomatous lesion. Lung involvement is rare. The radioclinical manifestations of this infection are polymorphic and confusing. The form revealed by a solitary pulmonary nodule is exceptional and not documented in the literature. *Case Presentation*. We report a case of a 71-year-old patient, 25-year package smoker, revealed by repeated moderate-abundance hemoptysis with a rare radiological image: in the form of a solitary pulmonary nodule located at the left chest base. The negativity of bacteriological research, endoscopic samples, the failure of treatment of the bleeding by medical and embolization, and at last the suspicion of a neoplasia led us to perform a thoracotomy for diagnostic and therapeutic purposes. This surgery highlighted evidence of filamentous basophilic grains posing the diagnosis of PA. Appropriate treatment allowed a cure without recurrence after 02 years of follow-up.

**Conclusion:**

Pulmonary actinomycosis can be revealed by different radiological forms. Pulmonary actinomycosis should be considered in the presence of any solitary lung nodule in order to reduce the morbidity and cost associated with thoracotomy.

## 1. Background

Pulmonary actinomycosis (PA) is a rare and ubiquitous bacterial disorder, combining subacute or chronic focal suppuration and an expansive fibrogranulomatous lesion [1]. Lung involvement is rare [2]. The radioclinical manifestations of this infection are polymorphic and confusing [3, 4]. Bacteriological diagnosis is difficult. Surgery in the absence of diagnosis has a diagnostic and therapeutic interest. We report a case of a 71-year-old patient, a 25-year package smoker, revealed by repeated moderate-abundance hemoptysis with a rare radiological image: in the form of a solitary pulmonary nodule located at the left chest base. The negativity of bacteriological research, endoscopic samples, the failure of treatment, and embolization led us to perform a thoracotomy for diagnostic and therapeutic purposes which highlighted evidence of filamentous basophilic grains posing the diagnosis of PA. Appropriate antibiotic treatment combined with surgical treatment allowed a cure without recurrence after 02 years of follow-up. PA must be considered before any solitary lung nodule (SLN) in order to decrease the morbidity related to the thoracotomy.

## 2. Observation

**Figure 1:**
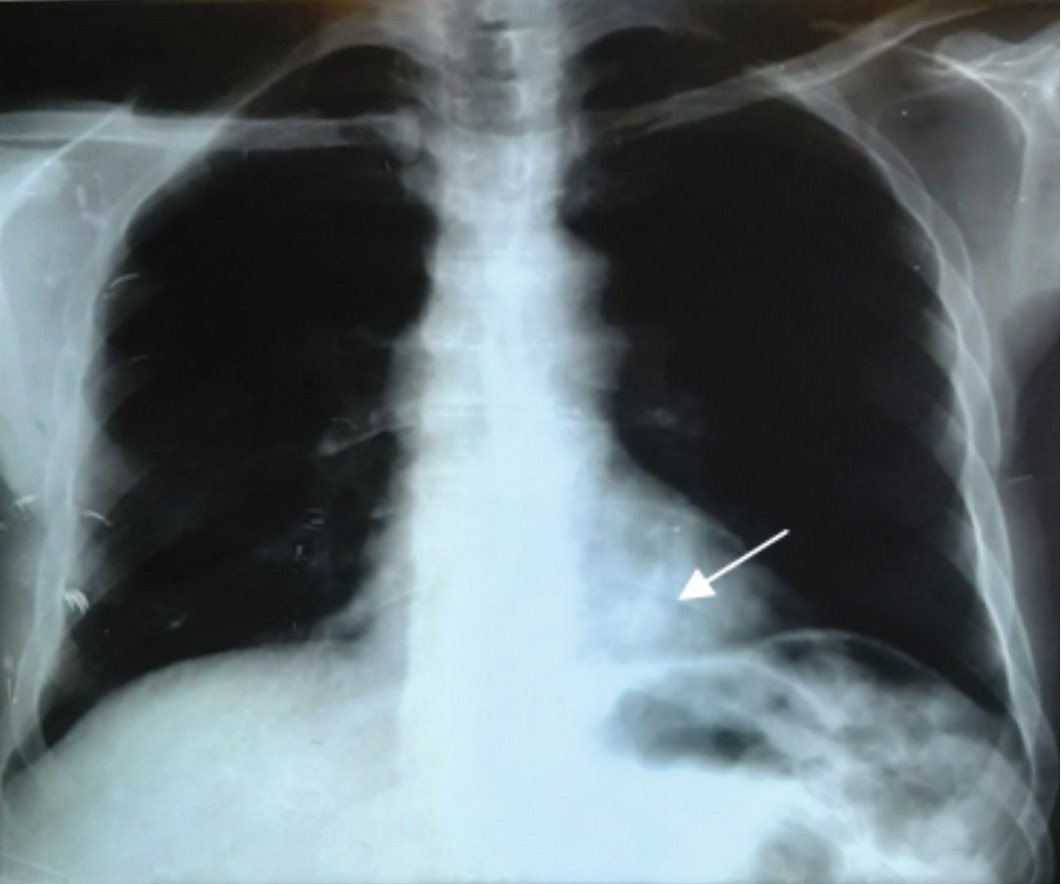
Chest X-ray face (solitary pulmonary nodule of the heart-phrenic angle).

**Figure 2:**
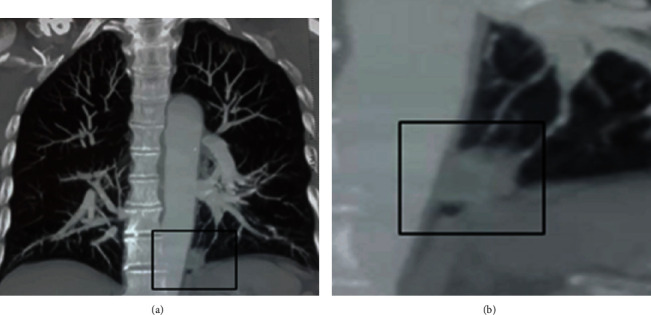
(a) Dense para-aortic pulmonary angiographic nodule. (b) Enlargement of the nodule.

**Figure 3:**
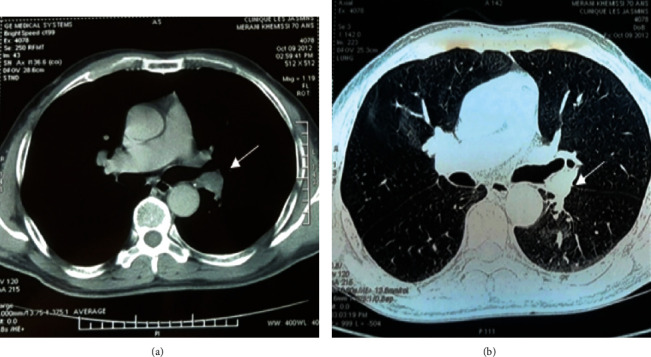
(a) A 3 cm dense para-aortic NPS. (b) Mediastinal window with a frosted glass appearance of hemoptysis.

The observed case is a 71-year-old man, 25-year package smoker, without alcoholic intoxication, and no infectious history. He was hospitalized in the pulmonary department of the University Hospital Center of Annaba, for the management of a moderate abundance of hemoptysis (recurrent of 50-100 ml on average per episode), evolving for 02 weeks. This hemoptysis was preceded two months previously by a cough with mucopurulent expectoration and pain in the left basithoracic side. The patient reports asthenia with loss of 8 kg of weight in 2 months. His general condition was preserved with slight skin pallor, a fever of 38.2°, an arterial pressure of 120/70, and saturation at 96% in ambient air. The physical examination and lung auscultation were unremarkable. The frontal chest X-ray showed a solitary pulmonary nodule (NPS), dense and rounded, 03 centimeters in diameter left basal retrocardiac (Figure 1). A pulmonary angiogram made in front of the chest pain and a level of D-dimer at 600 IU/ml eliminated a pulmonary embolism and objected a rounded dense opacity of 03 cm in left para-aortic diameter (Figures 2(a) and 2(b)). A chest scanner with injection of contrast product revealed a left inferior lobar para-aortic tissue mass of 03 cm in diameter, speculated with a homogeneous density, associated with an anterior frosted glass opacity and around the mass (would correspond to hemoptoic bleeding). The mediastinal window of the high-resolution chest thoracic scan does not find lymphadenopathy (Figures 3(a) and 3(b)). The search for tuberculosis in sputum was 03 times negative.

Bronchial fibroscopy with biopsy and cytology showed a nonspecific grade II inflammatory aspect and bacteriological and fungal research which was negative.

Anatomopathological study of the surgical specimen found a mass 3 cm in diameter, the site of a dense peribronchiolar inflammatory infiltrate. These were distended and ulcerated, the musculocartilaginous framework was replaced by fibrous tissue, and the bronchial lumen was filled with filamentous basophilic grains at the periphery, corresponding to grains of actinomycosis associated with lesions of hemorrhagic alveolitis and hyperplasia of the lymphoid follicles. This aspect supports the diagnosis of pulmonary actinomycosis. Preoperative and postoperative parenteral antibiotic therapy based on cephalosporin was instituted for 02 weeks, followed by amoxicillin 3 grams for 04 months.

## 3. Discussion

Pulmonary actinomycosis (PA) is a rare bacterial condition. Six species of Actinomyces are potentially pathological to humans, of which Actinomyces israelii is the most common. It is a gram-positive anaerobic bacterium that colonizes the oropharynx and gastrointestinal and genital flora [5]. Airway contamination occurs by aspiration of airway secretions. The pulmonary localization is favored by some local sites, such as chronic obstructive pulmonary disease, bronchiectasis, and cancerous tumors, or by risk factors such as alcohol consumption, diabetes, immunosuppression, and HIV [6]. Although recent publications show that 50% of PA can occur in patients without particular health problem, like our case [7]. PA occurs 3 to 4 times in men, aged 30 to 50 years [8]. However, it can be seen at an older age as is the case with our patient who was 71 years old at the time of diagnosis. PA is known for its radiological and clinical polymorphism, hemoptysis is the revealing symptom in 22 to 61% of cases, and this hemoptysis can be serious in 9% of cases [9]. Other respiratory symptoms may be associated with a type of cough with sputum. Three blood networks are involved in the mechanism of hemoptysis during actinomycosis: a bronchiolar network, a revascularization network, and a systemic arterial network, which is the most incriminated explaining the refractory character to medical treatment of hemoptysis and the use of embolization and therapeutic surgical treatment [10]. The diagnosis of PA is often late and exclusionary, and the radiological and clinical picture suggests initially pulmonary suppuration, tuberculosis, and especially bronchial cancer, which remains the most frequently mentioned diagnosis, explaining the high rate of diagnosis by thoracotomy [11]. The definitive diagnosis is bacteriological or histological. Detection of the germ in sputum, bronchial secretions, or bronchial lavage fluid is difficult due to the anaerobic character, commensal contamination, and frequent association with other organisms [12]. The specific, but not pathognomonic, histological appearance of actinomycosis is marked by the identification of yellowish filamentous grains on percutaneous, transbronchial, or surgical biopsies [13]. In other situations, the multiplication of bacteriological and histological samples by nonsurgical means improves diagnostic performance and morbidity [14]. In our patient, the bronchial samples were negative. Some authors have studied the contribution of the PET scan in the diagnosis of PA. However, this examination did not make it possible to make a clear difference between PA and malignant lesions [15]. The radiological forms of PA are either image of condensations sometimes excavated which can sit in any lobe. The right basal site as in our case is frequent and is linked to the etiopathogenic mechanism of colonization of the respiratory tract by aspiration. Pseudotumor images of masses or nodules leading to neoplasia can be seen [16]. Solitary pulmonary nodule (NPS) as a mode of radiological disclosure of PA is rare: It corresponds to an early stage of the disease, which in the absence of suitable treatment progresses to cavitation [17]. Associated with surgical treatment, the management of PA calls for antibiotic therapy based on Peni G in high doses (12 to 20 million units) with a first-line relay based on amoxicillin (3 g/24 h) for 3 to 6 months. Tetracyclines, macrolides, and vibramycin can also be used [7, 8]. Our patient was treated with amoxicillin 3 g/24 h for 03 months with good tolerance. The course under treatment is generally favorable. In our patient, at one-month follow-up, we observed a disappearance of asthenia, hemoptysis, cough, and sputum. Normalization of the biological assessment the 2-year imaging showed the total absence of recurrence.

## 4. Conclusion

Pulmonary actinomycosis can be revealed by rare radiological forms. Pulmonary actinomycosis should be considered in the presence of any solitary lung nodule in order to reduce the morbidity and cost associated with thoracotomy.

## Data Availability

The patient record with all screening elements is available at the Pulmonology Department of the University Hospital of Annaba, Algéria.
